# The oxytocinergic system in functional neurological disorder: Preliminary testing of associations with interoception and childhood trauma

**DOI:** 10.1016/j.cpnec.2026.100376

**Published:** 2026-08-08

**Authors:** Natascha Stoffel, Juan Ansede-Bermejo, Cristina Concetti, Ángel Carracedo, Selma Aybek

**Affiliations:** a Faculty of Science and Medicine, University of Fribourg, Switzerland; b Fundación Pública Galega de Medicina Xenómica, Centro Nacional de Genotipado, Santiago de Compostela, Spain; c Instituto de Investigación Sanitaria de Santiago (IDIS), Santiago de Compostela, Spain; d Center for Research in Molecular Medicine and Chronic Diseases (CiMUS), Universidade de Santiago de Compostela (USC), CIBERER-ISCIII, Santiago de Compostela, Spain

**Keywords:** *OXTR*, Salivary oxytocin, Methylation, Interoception, Childhood trauma

## Abstract

The oxytocinergic system has been proposed as a vulnerability factor in the pathophysiology of Functional Neurological Disorder (FND), a condition associated with stressful life-events and disturbed bodily awareness. Recent work from our group demonstrated reduced interoceptive function alongside elevated salivary oxytocin (OXT) levels in patients with mixed-FND.

Here, we tested the same patients with FND (N = 41) and matched healthy controls (N = 48) of that study, addressing two additional aims: (I) exploratively testing the genetic associations of the oxytocin receptor gene (*OXTR*) (i.e. investigating both genetic and epigenetic variables between groups and in interaction with interoceptive measures), and (II) evaluating whether integrating (epi)genetic and OXT measures improves overall model fit to explain interoceptive dysfunction and self-reported childhood trauma scores.

No group difference was observed regarding the allelic distribution of the rs53576 SNP or in *OXTR* methylation levels. For salivary OXT, an interaction effect between *OXTR* genotype and intron methylation (p = 0.036), as well as an interaction between group and genotype (p = 0.026) was identified. With respect to aim II, including oxytocinergic variables in the model did not explain additional interoceptive variance. However, including *OXTR* promoter methylation rates and salivary OXT levels alongside the group variable appeared to improve model fit and to account for more variance in self-reported childhood trauma.

While our preliminary genetic findings need to be interpreted with caution due to limited sample size and exploratory analyses, we suggest future research to profit from the inclusion of oxytocinergic markers when investigating risk-factors associated with FND.

## Highlights

• Salivary Oxytocin in FND may depend on rs53576 genotype.
• No overall genetic or epigenetic group difference at *OXTR*.
• Potential involvement of oxytocinergic variables in childhood trauma variability.

## Graphical abstract

**Figure:**
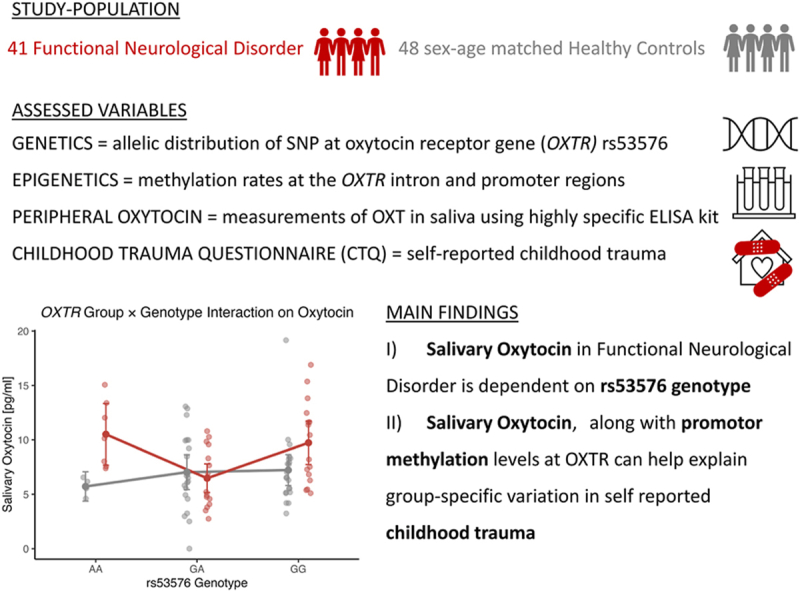

## 1 Introduction

The hormone and neuropeptide oxytocin (OXT) plays a crucial role in regulating autonomic, emotional, and behavioral processes, with specific context- and environment-dependent effects on the stress response system [1,2]. In addition, this system plays a central role in homeostatic control and interoceptive processing [3,4]. Interoception, the sensing of signals from within the body that maintain physiological homeostasis, has in its dysfunction been associated with a number of psychiatric disorders [5]. The oxytocinergic system may also be implicated in the consequences of early-life adversity, including traumatic experiences, suggesting its role as a potential biological mechanism underlying the development of an altered stress-response [[6], [7], [8]]. Recent evidence indicates that alterations within the oxytocinergic system may contribute to the pathophysiology of Functional Neurological Disorder (FND) [9], a disorder at the interface of neurology and psychiatry [10]. Besides FND being a common disorder, there is a lack of understanding regarding the underlying pathophysiology that results in a dysfunctional regulation of stress, bodily signals and FND symptoms [11]. Adverse life events such as childhood trauma or emotional neglect are recognized as a risk-factor for FND [12], but have also been shown to alter the oxytocinergic system [13,14].

Preliminary findings demonstrated a potential risk-allele for FND at the rs53576 SNP of the *OXTR*, and higher methylation rates at this *OXTR* in patients with FND compared to healthy controls [15]. While the latter result of epigenetic group differences has not been replicated by a larger study sample [9], a significant interaction of *OXTR* methylation with genotype on salivary OXT levels in FND has been identified in a more complex analysis. This points toward a disorder-specific modulation of the oxytocinergic system that might involve a compensatory upregulation of peripheral OXT, such as measured in saliva [9]. This notion is further supported by a following study in another sample of patients with FND, investigating salivary OXT while carefully controlling for potential covariates and demonstrating that salivary OXT concentrations are elevated in patients compared to controls [16]. Further, the levels of salivary OXT correlated negatively with self-reported interoceptive accuracy (IAS), highlighting its involvement in representing (internal) bodily awareness. Within the interoceptive system, OXT is discussed to increase the encoding of interoceptive signal precision and salience in the general population [17,18], and may also contribute to the attenuation of interoceptive input necessary for constructing a “generative self”, dependent on early life attachment experience [19].

What we here refer to as the oxytocinergic system is the interplay between genetic, epigenetic, and peripheral markers. For the genetics, this is in particular the two SNPs rs53576 and rs2254298 of the oxytocin receptor gene (*OXTR*). For the epigenetics, we here discuss methylation rates at both the promoter and the intron 1 region. Finally, for peripheral markers, we refer to measurements taken from saliva. Generally speaking, there has been evidence that the frequency of the A allele in rs53576 are associated with clinical phenotypes or hypermethylation [20], and more methylated *OXTR* would lead to less *OXTR* transcription [21,22]. However, methylation levels (at different sites of the *OXTR gene*) are differently associated with different clinical phenotypes, sometimes sex-specific [[23], [24], [25]]. For example, the *OXTR* promoter region has been associated with emotional neglect and depressive symptomatology in affective disorders [26] and implicated in anxiety in females only [24].

To date, there is no study that has investigated all of what we here call the oxytocinergic system along with interoceptive variables and childhood trauma in FND. A previous study in the same population as here analyzed has shown lower respiratory sensitivity in patients compared to healthy controls (HC) [27], as well as enhanced salivary OXT levels for FND associated with self-reported interoceptive accuracy (assessed via IAS) and a group-specific association with attachment insecurity (assessed via experience in close relationships (ECR) questionnaire) [16]. However, this study now further expands these findings with genetic and epigenetic analyses. Please find an overview of our studies and the associated populations discussed in the supplementary material, Table 1.

**Table 1:** Genomic regions and CpG sites included in methylation assays.

OXTR Gene Region	CpG Unit	CpG Site coordinates
Intron (chr3:8,810,749–8,810,920)	OXTR_2_CpG_2	chr3:8810874-8810875
	OXTR_2_CpG_5	chr3:8810832-8810833
	OXTR_2_CpG_6/7	chr3:8810807-8810808/chr3:8810797-8810798
	OXTR_2_CpG_8	chr3:8810774-8810775
Promoter (chr3:8,811,281–8,811,651)	OXTR_3_CpG_2	chr3:8811543-8811544
	OXTR_3_CpG_3	chr3:8811437-8811438
	OXTR_3_CpG_4	chr3:8811399-8811400
	OXTR_3_CpG_5/6	chr3:8811363-8811364/chr3:8811359-8811360
	OXTR_3_CpG_8	chr3:8811332-8811333

Note: CpG site coordinates with reference to genome version GRCh37/hg19.

For this study, we present two main aims. The first includes the analysis of oxytocinergic involvement in FND. We hypothesize that I) the presence of the A-allele in rs53576 at the *OXTR* acts as a vulnerability factor for FND, and that genotype impacts epigenetic oxytocinergic variables (i.e., methylation levels of the promoter and intron 1 regions). Then, we hypothesize that II) methylation levels at the *OXTR* are higher for patients compared to HCs, and III) that there is a group-specific interaction between genotype, *OXTR* methylation and salivary OXT. These analyses discuss previously identified associations from different samples [9,15]. The second study aim tests whether the addition of all these oxytocinergic variables would benefit the identified association with interoceptive variables (i.e., behavioural outcome of the respiratory resistance sensitivity task; RRST and of the self-reported interoceptive accuracy scale; IAS). We hypothesize that IV) the model of group interacting with genetic, epigenetic, and peripheral markers of the oxytocinergic system would better explain interoceptive variance. Finally, we hypothesize that V) the inclusion of the oxytocinergic system in the model would help explain the observed variability in interoceptive variables. Finally, the same approach of testing model fit to explain the group-specific variance in the self-reported childhood trauma score (CTQ), as a key risk factor for FND, will be applied. These analyses complement previously identified associations from the same sample [16,27].

## 2 Methods

### 2.1 Participants

A total of N = 92 adults (aged >18) were originally enrolled in the study investigating the oxytocinergic and interoceptive system in a mixed cohort of patients with FND and sex-aged matched healthy controls (HC) [16,27,28]. Sample size estimation was based on prior data examining salivary cortisol in FND and HC [29] – as the primary aim was the investigation of salivary OXT - and required a total of N = 89 participants. For the present analyses, three patients with FND were excluded due to missing data at the (epi)genetic level. Hence, a total of N = 89 (N_FND_ = 41 and N_HC_ = 48) were included in the following analyses, with two further exclusions due to missing data for all analyses involving salivary OXT N = 87 (N_FND_ = 40 and N_HC_ = 47). Patients diagnosed with FND by a certified neurologist (ICD-11: F44.4–44.7) were able to participate, recruited from two Neurology clinics in Switzerland; the University Hospital/Inselspital Bern, and the Cantonal Hospital of Fribourg (HFR). HCs were recruited via flyers, word-of-mouth, and online advertisements. Exclusion criteria for both groups were (a) severe comorbidity such as psychosis or major depression (b) brain surgery (c) substance abuse (d) cardiovascular disorders, and (e) pregnancy/breastfeeding (verification was conducted via urine test for females of childbearing age). The study was carried out at the HFR and was approved by the local Ethics Committee of the Canton Bern (2023-00469), as well as registered at clinicaltrial.gov (NCT06084325). The study was conducted according to the Declaration of Helsinki, and all participants provided informed consent prior to the study.

### 2.2 Psychometric assessment

Beck's Depression Inventory (BDI-II; Beck et al., 1996 [30]) and the State-Trait Anxiety Inventory (STAI; Spielberger et al., 1970 [31]) were used to test affective symptoms, whereas a composite sum score would be used in the analysis to test for these potential covariates. The Interoceptive Accuracy Scale (IAS; Murphy et al., 2020 [32]) was used to assess interoception at the level of self-report. Respiratory sensitivity was assessed using the respiratory sensitivity resistance task (RRST; Nikolova et al., 2022 [33]). Previous publications discuss the initial assessment of these and further variables between groups, along with the rationale for the choice and the interpretation of the results [16,27,28]. The respiratory sensitivity outcome was chosen for further investigation with the oxtocinergic variables as it did show a significant group difference [27], and the self-reported IAS was already shown to be correlated with salivary OXT [16], warranting further investigation by the addition og (epi)genetics.

### 2.3 Salivary oxytocin

Salivary OXT was assessed as an average from four timepoints, designed to account for fluctuation throughout the day and in response to food intake or cognitive/physical activity. The setup of the study design and the discussion of results on salivary OXT is available in a previous publication [16].

### 2.4 DNA samples and quantification

For each participant, a total of 15 ml of peripheral blood was collected using two 7.5 mL EDTA S-Monovette tubes (Sarstedt, Nümbrecht, Germany) and frozen at −20°C until its use. DNA samples were extracted according to the manufacturer's protocol using the QIAmp DNA Blood kit (Qiagen, Hilden, Germany). For each sample, DNA concentration was quantified using Quant-it dsDNA Broad-Range Assay Kit (Invitrogen, Thermo Fisher Scientific, MA, USA) according to the manufacturer's protocol. The samples were normalized at 20 ng/μL and were stored at −80°C.

Genotyping and methylation analysis were carried out at *Centro Nacional de Genotipado* (CEGEN) located in the facilities of *Fundacion Pública Galega de Medicina Xenómica* (Santiago de Compostela, Spain).

### 2.5 Genotyping analysis

Genotyping assay was designed using Assay Designer 4.0 software (Agena Bioscience, San Diego, CA, USA) to obtain the sequence of primers used in the genotyping of SNPs rs53576 and rs2254298 located within *OXTR* gene. The rs53576 and rs2254298 SNPs were genotyped in a single multiplex assay. Details on the sequence of PCR and SBE primers employed in genotyping assays can be found in Supplementary Material (Table S1).

### 2.6 EpiTYPER methylation analysis

Quantitative DNA methylation analysis was performed through EpiTYPER assay employing the MassCLEAVE chemistry, a bisulfite-treatment-based method for detection and quantitation of DNA methylation [34] using base-specific cleavage and matrix-assisted laser desorption/ionization time-of-flight mass spectrometry (MALDI-TOF MS) integrated into the MassARRAY system (Agena Bioscience, San Diego, CA).

### 2.7 CpG site selection

Selection of candidate CpG sites (Table 1) located within the intron 1 region of the oxytocin receptor (*OXTR*) gene was selected based on previous findings in patients with FND [9,15]. Further, we also included CpG sites located within the promoter region of *OXTR,* as this genetic region has previously been shown to be of relevance for the phenotypes of anxiety in the female sex [24].

### 2.8 Flanking sequence and primer design

Flanking sequences for each selected CpG were retrieved from the UCSC genome browser (https://genome.ucsc.edu/) using the human genome assembly (GRCh37/hg19), and selecting the region of interest with 300 base pairs (bp) upstream and downstream of the target CpGs. Primer design for EpiTYPER assays was performed using Agena Bioscience EpiDesigner software (http://www.epidesigner.com). The specificity of selected primers was tested *in silico* through the software BiSearch [35] available at http://bisearch.enzim.hu/. Sequence of PCR primers employed in methylation assay can be found in Supplementary Material, Table S2.

### 2.9 Bisulfite conversion samples

Bisulfite conversion was performed with 0.5 μg of genomic DNA using the EZ 96-DNA methylation kit (Zymo Research, Orange, CA, USA) following the manufacture's guidelines. For quality control, all samples were analyzed in duplicates and four internal controls were included in every plate reaction: a human high methylated DNA control (EpigenDx Inc.); a human low methylated DNA control (EpigenDx Inc.); a nontemplate DNA control; and a non-transformed DNA control.

### 2.10 Methylation assays

Each PCR reaction was set up in 384-well microplates according to the following conditions: 2 μL of converted DNA in a final volume of 5 μL containing 0.42 μL of HPLC-grade water, 0.5 μL of PCR buffer (10x), 0.04 μL of dNTPs (25 nM), 0.04 μL of PCR enzyme (5U/μL) and 1 μL of each primer forward and reverse (1 mM). The thermal cycling conditions for the reaction consisted of an initial denaturation step at 95°C for 10 min, followed by 4 cycles of 95°C for 20 s, 62°C for 30 s and 72°C for 1 min, followed by 4 cycles of 95°C for 20 s, 58°C for 30 s and 72°C for 1 min, followed by 38 cycles of 95°C for 20 s, 54°C for 30 s and 72°C for 1 min followed by a final extension step of 72°C for 3 min.

Excess nucleotides were removed with 1.7 μL of RNase-free ddH2O and 0.3 μL of Shrimp Alkaline Phosphatase (SAP; 1.7U/μL) to the full PCR volume, then incubated at 37°C for 20 min and 85°C for 5 min. After quality checks of the amplification bands in Agilent 4200 TapeStation system (Agilent, Germany) with the D1000 Kit according to the manufacturer's information. Reverse transcription/RNase A cleavage reactions were performed with the following conditions: 3.21 μL of RNase-free ddH2O, 0.89 μL of T7 polymerase buffer (5x); 0.22 μL of T cleavage mix, 0.22 μL of DTT (100 mM); 0.4 μL of T7 RNA & DNA polymerase (1.7U/μL) and 0.06 μL of RNase A in 3 μL of PCR-SAP product, incubated at 37°C for 3 h.

Cleavage reaction products were desalted by adding the clean resin following the manufacturer's protocol (Agena Bioscience). Desalted products were dispensed onto a 384 SpectroChip II™ using the MassARRAY system with Chip-Prep Module (CPM) and spectra were acquired using the MA4™ mass spectrometer (Agena Bioscience, San Diego, CA). As all samples were PCR amplified and run on EpiTYPER in duplicate, mean methylation levels were averaged from replicates, discarding those results for which the standard deviation between replicates was higher than 10%.

### 2.11 Statistical analyses

Part I (Hypothesis I-III) attempts to test the previously discussed effects of genotype, methylation, and salivary OXT [9,15,36] in a new cohort. For that, genotypic data for two single nucleotide polymorphisms (SNPs) within the oxytocin receptor gene (*OXTR*), rs2254298 and rs53576, were analyzed using the setupSNP() function in R. Hardy-Weinberg Equilibrium (HWE) tests were used to identify deviations from expected genotype distributions, which may suggest issues such as genotyping errors or population stratification. Our results of *p* > 0.05 indicated no deviation from HWE. Logistic regression models were fit using WGassociation() and then specifically for the co-dominant model, with and without adding covariates (age, sex, CTQ total, and anxiety-depression sum scores). The results of the recessive model can be found in the Supplements, Table S1. Further, an ANOVA tested the interaction of oxytocinergic variables and group regarding salivary OXT. Genotype-specific association to both salivary OXT and OXTR methylation rates was then exploratively tested using Kruskal-Wallis test, illustrating the genotype and group association descriptively. In these analyses, genotype is assessed as a categorical variable (representing again a codominant model that does not assume dominance or additive effects of any allele).

Part II (Hypothesis IV and V) attempts to explore the effect of including (epi)genetic variables in the models that may explain interoceptive group differences. Here we build on previously established findings [16,27] and investigate the benefit of adding (epi)genetic data. For that, the factor of group and all oxytocinergic variables were included in a four-way interaction ANOVA, assessing their effects on the established interoceptive dysfunction, i.e., respiratory sensitivity via RRST [27] and self-reported accuracy scores via IAS [16]. We report the overall model fit, instead of interpreting the individual interactions. We also tested whether the oxytocinergic system is involved in the variance around the experience of childhood trauma. In case of an improved model fit from the addition of oxytocinergic variables, we will use the linear models to guide interpretation concerning the direction of effects of the included variables. Visualization of these variables (RRST, IAS, CTQ) in regard to the oxytocinergic system (salivary OXT and both methylation levels of *OXTR*) can be seen in the Supplementary material.

Please note that CTQ scores were used differently across the two analytic parts according to the respective research question. In Part I, CTQ total was included as a covariate given that childhood trauma is a prominent risk factor in FND and may confound group differences when testing biological and clinical variables. In Part II, CTQ was further explored as an outcome variable using oxytocinergic variables and testing the improvement for model fit. This analysis was exploratory and non-directional, examining the biological role of this risk factor in FND, which does not test causal or temporal pathways.

Given the number of statistical models tested across Parts I and II, and because the present analyses were intended to explore previously hypothesized oxytocinergic associations in FND, rather than to provide evidence for a single primary endpoint, we treated the analyses as exploratory and rather hypothesis-generating. Therefore, we want to highlight that no global family-wise correction was applied across all ANOVAs, Kruskal-Wallis tests, logistic regression models, and model-comparison analyses. Consequently, p-values should be interpreted cautiously, particularly for effects close to the conventional significance threshold.

Finally, sensitivity analyses in the medication-free subsample, as well as sex stratified analyses, are reported in the Supplementary material.

## 3 Results

### 3.1 Demographics

Our sample is matched in sex and age, while depression, anxiety, childhood trauma scores, and intake of medication are higher in the patient group. Table 2.

**Table 2:** Demographics.

Variable	Overall, N = 89	HC, N = 48	FND, N = 41	Test statistics (df)	Effect size [Confidence Interval]	p-value
Sex: count female (%)	65.0 (73.0)	35.0 (72.9)	30.0 (73.2)		OR = 0.99 [0.34, 2.79]	>0.9
Age: mean years (SD)	37.9 (12.4)	37.8 (13.0)	38.1 (11.8)	t = −0.10 (87)	d = −0.02 [-0.44, 0.40]	>0.9
Psychotropic Medication: count (%)	21.0 (24.1)	2.0 (4.3)	19.0 (46.3)		OR = 18.37 [3.89, 176.40]	<0.001
Depression: BDI-II median score (IQR)	8.0 (12.0)	4.0 (7.0)	15.0 (14.0)	W = 285	d = −1.38 [-1.84, −0.91]	<0.001
Trait Anxiety: STAI-T mean score (SD)	41.6 (11.7)	37.3 (10.3)	46.7 (11.2)	t = −4.10 (82)	d = −0.88 [-1.31, −0.44]	<0.001
Childhood Trauma: CTQ median score (IQR)	36.0 (25.0)	34.0 (12.3)	46.0 (27.0)	W = 615	d = −0.67 [-1.09, −0.24]	0.002

Note: To calculate the group differences, for binary data, fisher tests were used – reporting the count (%), for numeric data with parametric distribution, Welch t-tests were used - reporting mean (SD), and for non-parametric distribution, Wilcoxon rank-sum tests were used – reporting median (IQR).

### 3.2 Genotyping analysis

In our data, for both rs2254298 and rs53576 the G was the major allele, with A being the minor allele, and a major allele frequency of 85%, and 65.6%, respectively. The co-dominant model was tested for allelic distribution, assuming no dominance and additive effects. None of the models reached significance (*p* > 0.05 for both SNPs). Table 3.

**Table 3:** Genetic association of oxytocin receptor gene (*OXTR*).

SNP ID	Model	Genotype	Cases		OR [95% CI]	*P*-value
			FND, N = 41 (46%)	Controls, N = 48 (54%)		
rs53576	Co-dominant	G/G	16 (39.0%)	23 (47.9%)	1.00	0.334
		G/A	17 (41.5%)	21 (43.8%)	1.51 [0.50, 4.57]	
		A/A	8 (19.5%)	4 (8.3%)	3.43 [0.65, 18.10]	
rs2254298	Co-dominant	G/G	32 (78.0%)	33 (68.8%)	1.00	0.723
		G/A	7 (17.1%)	14 (29.2%)	0.60 [0.17, 2.17]	
		A/A	2 (4.9%)	1 (2.1%)	1.19 [0.09, 15.51]	

Abbreviations: OXTR: Oxytocin receptor, OR = Odds Ratio, FND = Functional Neurological Disorder.

Models were calculated including covariates age, sex, CTQ total and affective sum score. The co-dominant model does not make any assumption of dominance or additive effects and considers all three genotypes as different categories.

### 3.3 Methylation analysis

For both the promoter and intron 1 region of *OXTR*, we calculated an average across the different CpG sites, while additionally analyzing each site separately.

**Fig. 1:**
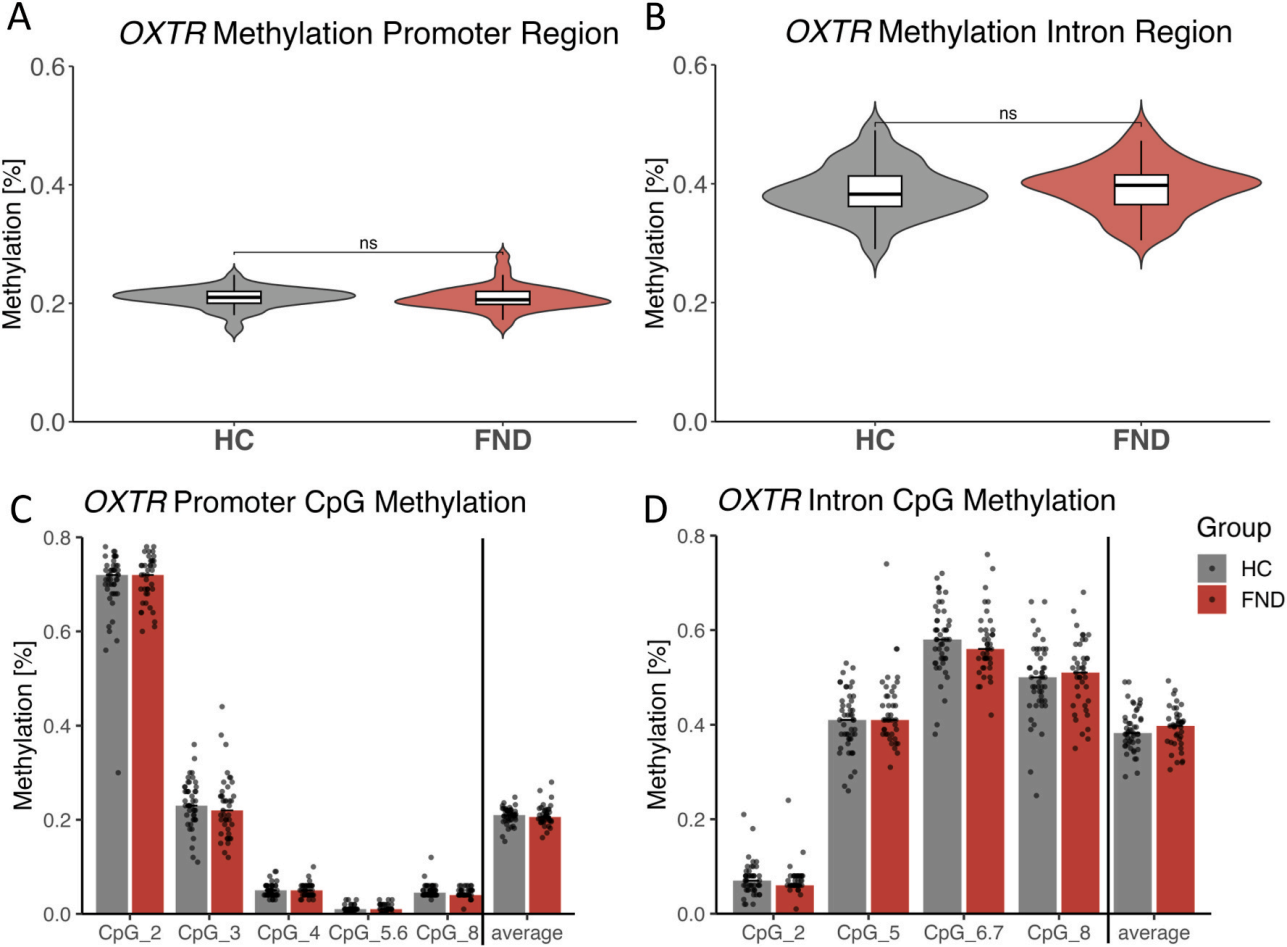
**Illustration of group differences in OXTR methylation rates at both the promoter and the intron Promoter regions**. N = 41 patients with Functional Neurological Disorders (FND) are compared to N = 48 Healthy controls (HC) using a Wilcoxon rank-sum test for both the A) average rates for Promoter and B) Intron region, as well as site-specific comparison testing each of the CpG sites individually with false discovery rate correction for multiple comparisons (C and D, respectively). Bars represent median methylation levels for each group and individual data points are overlaid as jittered dots. No significant group difference was detected.

On average, there is no difference between the groups for the intron region (p = 0.406) nor the promoter region (p = 0.439) using the Wilcoxon test for non-normally distributed data (Fig. 1A and B). Looking at each CpG site separately and correcting for multiple comparisons using FDR, there is no significant group difference for either of the specific sites (Fig. 1C and D).

### 3.4 Interaction model for OXT

**Fig. 2:**
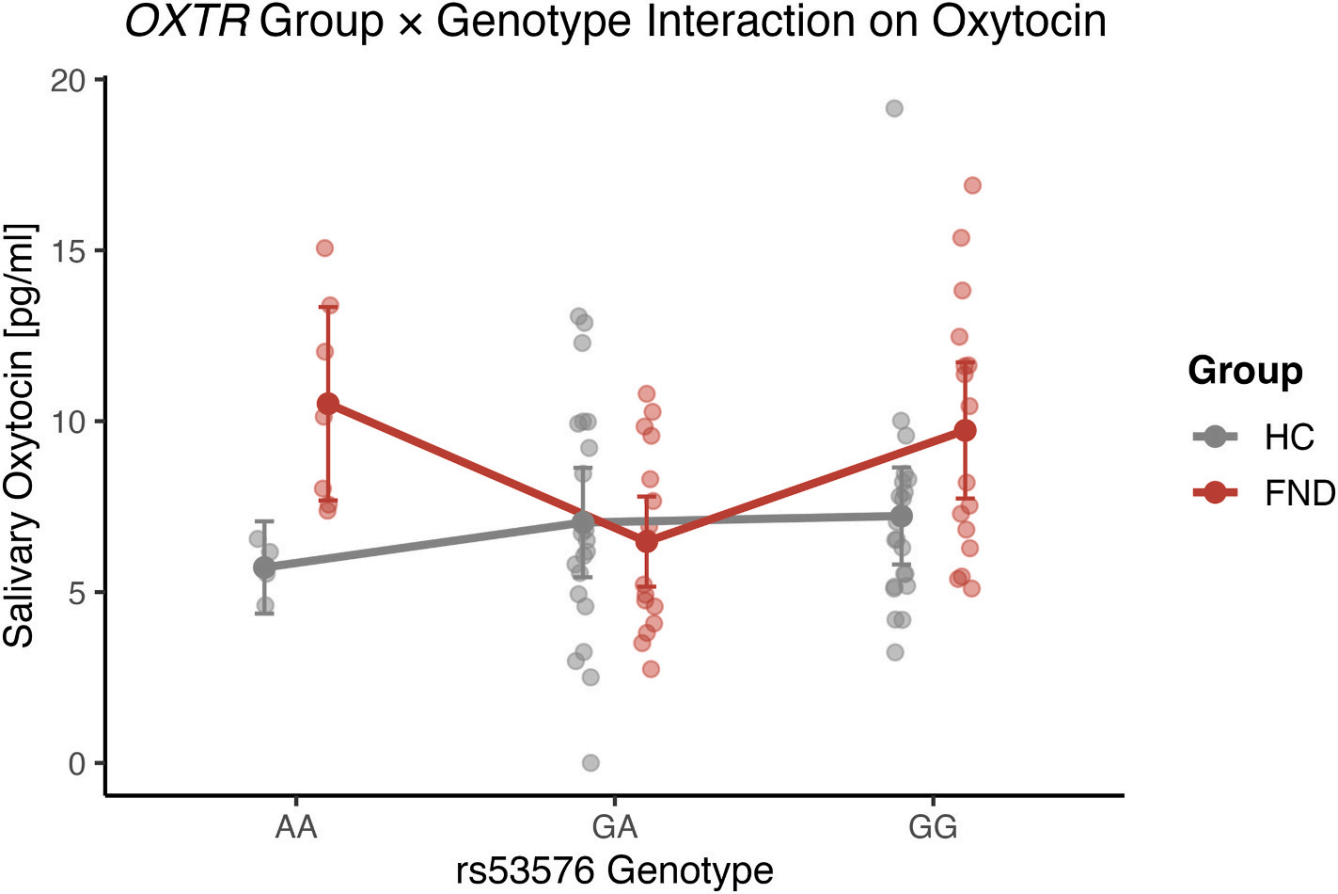
***Oxytocin dependent on genotype for patients with FND****. Within the N = 47 Healthy Controls genotype is distributed as follows: N*_*AA*_ = *4, N*_*GA*_ = *21, N*_*GG*_ = *22 and does not have an effect on salivary Oxytocin, while within the N = 41 patients with Functional Neurological Disorders (FND), genotypes are distributed as follows: N*_*AA*_ = *7, N*_*GA*_ = *17, N*_*GG*_ = *16, leading to a group × genotype interaction using an ANOVA: (F (2,72) = 3.84, p = 0.026), next to a main effect of group (p = 0.034).* Each dot represents a participant's salivary Oxytocin level, color coded based on genotype. The lines connect the group means (±95% confidence intervals) for each rs53576 genotype across the two groups. *Note that two participants (one patient with FND and one HC) were excluded from the analyses including salivary OXT due to missing data.*

Running a three-way interaction model for salivary OXT, a main effect of group (F (1,72) = 4.65, p = 0.034, partial η^2^ = 0.061) was identified, next to the two-way interactions between genotype and *OXTR* intron methylation (F (2,72) = 3.47, p = 0.036, partial η^2^ = 0.088) and between group and genotype (F (2,72) = 3.84, p = 0.026, partial η^2^ = 0.096). Visualization in Fig. 2, whereas interpretation must be done cautiously given the only nominally significant interactions derived from the modest sample size (i.e., genotype cells of AA n = 4-7) and the exploratory nature of this analysis. Follow-up comparisons using estimated marginal means (EMMs) were done to test the group interaction, comparing simple effects of group within each genotype. These analyses illustrated that only GG carriers exhibited a significant group difference, with patients with FND showing higher OXT levels than comparable HCs (EMM difference = −2.70, SE = 1.29, *t*(72) = −2.10, *p* = 0.040, 95% CI [−5.27, −0.13]). Examining then the simple effects of genotype within each group, there was an effect of genotype only in the FND group, namely for GA carriers exhibiting lower salivary OXT than GG carriers (difference = −3.46, SE = 1.13, *t*(72) = −3.07, Tukey adjusted *p* = 0.008, 95% CI [−6.15, −0.76]). For an overview of cell size per genotype and group, please see Supplementary Table 4.

### 3.5 Genotype-stratified group comparisons

In addition to the formal interaction model, we conducted stratified Kruskal-Wallis tests to describe and visualize salivary OXT and *OXTR* methylation (separately per promoter and intron region), stratified by group and comparing rs53576 genotypes. This should be understood as exploratory post-hoc decomposition of the previously discussed interaction findings.

**Fig. 3:**
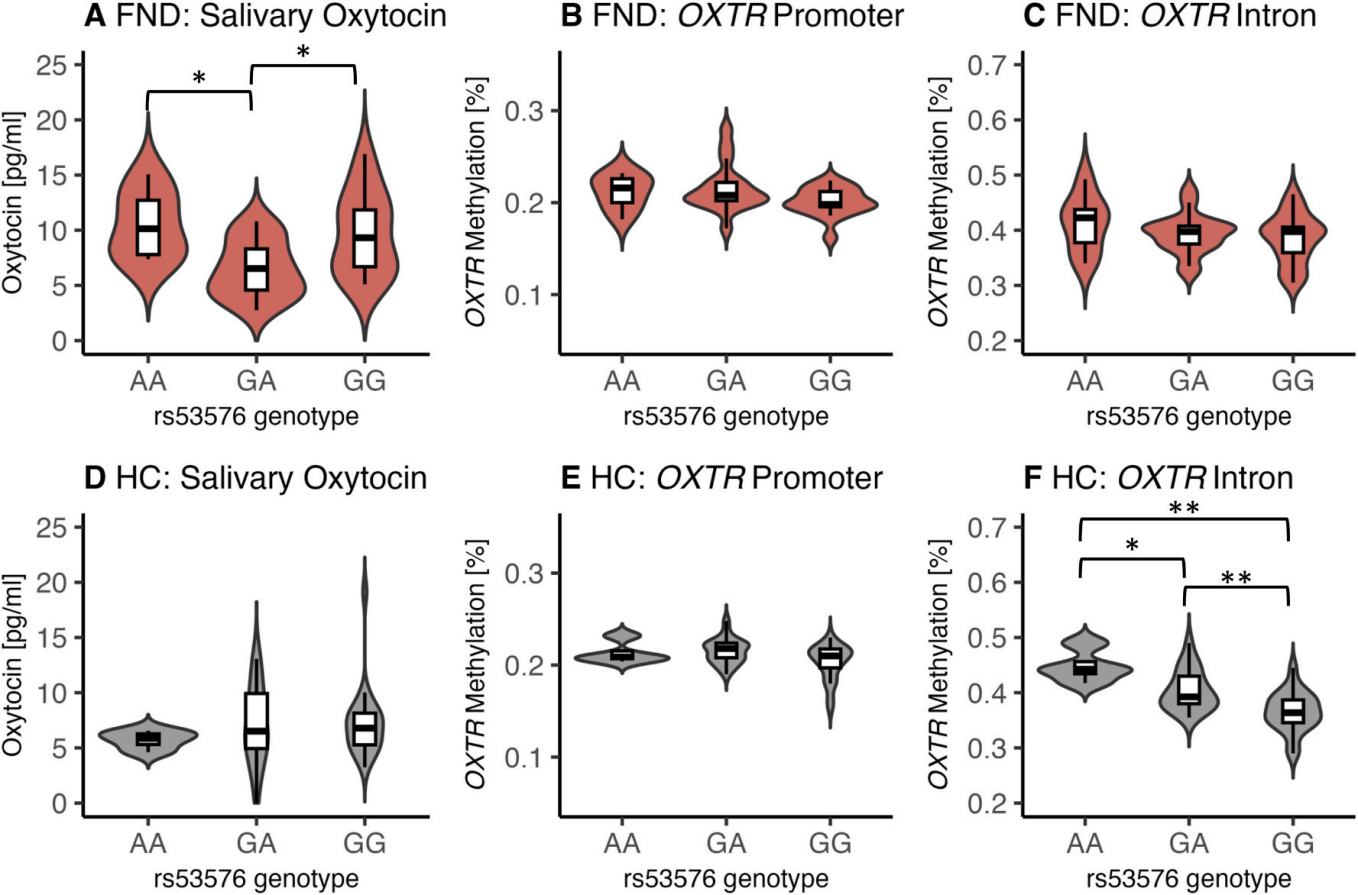
**Descriptive illustration of salivary Oxytocin and OXTR methylation rates in both the promoter and the intron regions, dependent on rs53576 genotype and shown separately per group**. A-C illustrates N = 40 patients with Functional Neurological Disorders (FND), and D-F illustrates N = 47 Healthy controls (HC). Genotype effects across the full sample were tested using Kruskal-Wallis tests, followed by post-hoc pairwise Wilcoxon rank-sum tests between genotype groups with false discovery rate correction for multiple comparisons. Levels of significance: * = p < 0.05, ** = p < 0.01, derived via paired Wilcoxon test, and correcting for multiple comparison using False Discovery Rate. Note that two participants (one patient with FND and one HC) were excluded from the analyses including salivary OXT due to missing data.

For patients with FND, salivary OXT differed between rs53576 genotypes (*χ*^*2*^(2) = 10.23, *p* = 0.006, FDR-adjusted *p* = 0.018), suggesting a genotype effect on salivary OXT in this group. Post hoc tests showed that GA carriers had lower oxytocin levels than both AA (*p* = 0.009, FDR-adjusted *p* = 0.013) and GG genotypes (*p* = 0.007, FDR-adjusted *p* = 0.013), while the difference between AA and GG was not significant (*p* = 0.492, FDR-adjusted *p* = 0.492). Fig. 3A.

For healthy controls, we identified a difference in *OXTR* intron CpG methylation between rs53576 genotypes (*χ*^*2*^(2) = 15.26, *p* = 0.001, FDR-adjusted *p* = 0.003), indicating genotype-related epigenetic variation in this subgroup. Post-hoc comparisons revealed that individuals with the GG genotype had higher intron methylation levels compared to both AA (*p* = 0.0009, FDR-adjusted *p* = 0.003) and GA carriers (*p* = 0.003, FDR-adjusted *p* = 0.004), while the difference between AA and GA was also significant (*p* = 0.045, FDR-adjusted *p* = 0.045). Fig. 3F.

### 3.6 Exploring model fit by adding oxytocinergic variables

For Part II, we tested whether the addition of oxytocinergic variables contributed to the previously established group differences in interoception, i.e., testing model fit using hierarchical linear regressions for both respiratory interoceptive sensitivity (RRST) and self-reported interoceptive accuracy score (IAS). For respiratory sensitivity, the base model including only group explained a small but significant proportion of variance (adjusted R^2^ = 4%, p = 0.031). Adding salivary OXT, *OXTR* intron or promoter methylation, as well as rs53576 genotype did not increase model fit. Information-theoretic indices (AIC/BIC) also favored the simplest model including only group. For IAS, the base model was already including salivary OXT based on established findings [16], demonstrating significant but modest effects (adjusted R^2^ = 14%, p = 0.001). Yet, neither the addition of methylation scores nor genotype improved explanatory power, and again AIC strongly favored the base model. Together, these results indicate that the addition of oxytocinergic variables did not account for more explanatory variance in either behavioral or self-reported indicators of interoception.

Concerning the risk factor of FND of experiencing childhood trauma, which has been associated with the oxytocinergic system [13,14], the same model fit analysis was conducted using childhood trauma as a predicted variable. Across all candidate models for explaining the variance in self-reported scores in CTQ, the model of *OXTR* promoter methylation, along with group, salivary oxytocin (and covariates of no interest; sex, age, affective symptoms) showed the best overall fit with the lowest AIC (743.1), and explained most variance, with adjusted R^2^ = 25.9%. It outperforms the smaller model of only adding salivary OXT (ΔF(1, 80) = 8.09, p = 0.006). In this model, *OXTR* promoter methylation emerged as a significant negative predictor, β = −261.09, p < 0.01, indicating that each percentage point of higher *OXTR* promoter methylation was associated with approximately 2.61 points lower CTQ total score. In other words, one standard deviation of higher *OXTR* promoter methylation was associated with the self-reporting of approximately 5.1 points lower CTQ total score. Further adding rs53576 genotype did however not improve model fit (ΔF(2, 78) = 0.81, p = 0.449, adjusted R^2^ = 25.6%).

## 4 Discussion

### 4.1 No difference in rs53576 genotype or *OXTR* methylation levels

For Part I, we attempted to test the (epi)genetic involvement around the oxytocinergic system in a new cohort, which could not identify a risk allele at the *OXTR* for FND (unlike a previous study sample [9,36]. As for not having adequate power from sample size, we are not able to conclude a final interpretation upon this finding. However, we present consistent and accumulating evidence of seeing no difference between groups regarding methylation [9].

### 4.2 Impact of genotype on salivary OXT in FND

We identified an interaction of genotype and group regarding the salivary OXT (Fig. 2), which was further described in Fig. 3A. Here too, caution is warranted for interpretation due to the nominal significance and small sample size regarding genotyping results (see also the Method section ‘Statistical Analysis’). However, from these preliminary results we could interpret that genotype acts as a potential vulnerability for an increased salivary OXT in FND. While it is normal that salivary OXT measures increase in response to situations of parental care, running or sexual self-stimulation [37,38], there has also been an involvement of genetic factors to moderate these changes [37,39]. In FND however, salivary OXT levels are elevated independently of such OXT-stimulating contexts [16], raising questions regarding the mechanisms underlying this increase. One possible explanation could be this interaction with genetic factors, suggesting a genotype-dependent alteration that may contribute to a stabilization of elevated OXT in FND irrespective of context. This hypothesis is particularly relevant given the established role of OXT in anxiolytic and stress-regulatory processes, as well as in facilitating active coping strategies [2,40]. Higher increases of OXT through the day were indicative of a more reactive cortisol release in children with autism [41]. Also, salivary OXT release of infants would increase in response to experiencing insensitive care from the mother, further interacting with carrying the G allele [37]. A similar interaction may happen for patients with FND in stressful experiences, which are more common in the life history of FND. Consequently, OXT-dependent active coping mechanisms, including enhanced muscle activation through increased oxygen consumption [2] might become chronically engaged and potentially contribute to the display of FND symptoms, such as involuntary movement. This could represent a preliminary hypothesized pathway linking genetic vulnerability and altered stress responsivity to FND symptom onset. However, this proposed mechanism spans distinct biological compartments without having assessed the individual links in this chain. Consequently, research is needed to investigate this hypothesized mechanism beyond the group-genotype interaction observed here.

### 4.3 Discussing a potential interaction model of OXT

There was no three-way interaction that points towards the involvement of a group-specific effect of (epi)genetic markers concerning salivary OXT, however, some interactions and main effects still indicate a complex interplay, highlighting the potential importance of the oxytocinergic system. We identified a nominal interaction between genotype and methylation rates at the intron 1 region – similar to previously reported [9] but independent of group. While of exploratory nature due to the limited sample size, this could suggest that there is an interplay between genetics, epigenetics and peripheral release of OXT in general. This is relevant because we need to consider this interplay and potential dependencies when discussing biological risk factors and, in the future, potential treatment avenues. Unfortunately, there are yet only a few studies that investigate both genotype and salivary OXT, and none that have done it in the same methodology as we did. Still, previous findings suggest that salivary OXT might not be dependent on the rs53576 genotype [42,43]. Hence, while this could indicate a false positive effect in our sample, we also want to point out the different analyses conducted, such as the use of a different analysis kit for salivary measurements (EIA vs ELISA method for OXT analysis, using extracted vs unextracted samples in saliva, calculating a recessive vs. a co-dominant genetic model for allelic distributions). Also, it should be highlighted that our samples for methylation analysis were taken from blood, which may limit our findings that the methylation levels are rather for tissue in the periphery, and might not represent the levels in the brain [44]. Similarly for the peripheral OXT measures, sampled via saliva; these values seem to correlate with the levels found in the brain [45], but it is not sufficiently clear whether our discussed peripheral level represents the OXT-concentration found in the brain.

### 4.4 Illustrating the genotype-stratified group comparisons

We also described that there might be a dependency on genotype for the methylation levels at the intron 1 region for HCs (Fig. 3F), which is a similar to what we have before identified in another cohort of patients with FND [9]. The AA-genotype has been associated with higher methylation levels in the presence of an affective disorder [20]. We conclude that the genotype-dependent methylation might be group independent, as this result appeared in different study populations (i.e., in HCs here, while in FND in Ref. [9], and in affective disorders in Ref. [20]). Having two A-alleles might represent a general biological mechanism of having higher methylation rates at the *OXTR* intron. Regarding our other findings, it is additionally the interaction between genetics and epigenetics that might be relevant for the stress-reactive OXT system within a specific context [2], and not the main effects.

### 4.5 No better model fit for adding oxytocinergic variables to interoceptive outcome

For Part II, we investigated the association between previously identified interoceptive markers in FND (such as lower respiratory sensitivity [27] and higher salivary OXT correlated with lower interoceptive self-report [16] with the newly added the (epi)genetic involvement of the oxytocinergic system. However, these previously discussed findings were not better explained by the addition of (epi)genetic variables, indicating that the interoceptive dysfunction in FND does not seem to be better explained by the oxytocinergic system in addition to group (and salivary OXT).

### 4.6 Better model fit for adding oxytocinergic variables to childhood trauma score

However, when we focus on the variability of the risk factor of childhood trauma, we see an increased model fit when adding promoter methylation levels, along with the variables group and salivary OXT. These results suggest that the dynamic oxytocinergic system (i.e., peripheral release in saliva and epigenetic modulation of methylation rates) may somehow be linked to the experience of childhood trauma. Our approach to test for model fit by adding additional variables is no causative test. However, knowing that both salivary OXT and methylation levels can change depending on stressful experience [1,2,46], oxytocinergic change may be a consequence of trauma. Yet, a reverse or a third-variable explanation cannot be excluded (e.g. shared genetic liability, current affective state influencing both retrospective trauma reporting and peripheral OXT). Literature suggests that experiencing early life adversities and anxiousness has been related to changes in methylation levels in the promoter region, in particular for females [24]. An interaction between lower *OXTR* promoter methylation and history of abuse predicted higher psychopathology, such as depressive and anxiety symptoms, in a large cohort of 393 adults characterized by their high psychosocial stress and experiences of violence [47]. Notable, FND is characterized by high psychosocial stress and reporting experiences of trauma [12], as well as being predominant in the female sex [48]. OXT seems to be highly relevant regarding coping with and responding to both physiological and psychological stressors, and may buffer the maladaptive response to stressful, traumatic experiences [1,6,8]. A study of 846 individuals with an affective disorder discussed non-significant association between *OXTR* promoter methylation and affective symptoms and childhood trauma, which may partly be because both genetic and peripheral measures were ignored, and the focus was merely on the methylation levels [26]. Finally, knowing how stressful and adverse life experiences can impact the development of the brain, or learned sensory processing [49], the oxytocinergic system emerges as a compelling candidate for understanding FND vulnerability and interoceptive dysfunction when considering the full system in its complex interactions.

At this point, an additional consideration should be discussed regarding the interpretation of the results derived from the here investigated FND patient group. Patients who underwent this study protocol that involved salivary assessment and blood withdrawal may represent a specific subgroup, and group-level associations may therefore obscure meaningful inter-individual differences. In this context, the previously discussed elevated salivary OXT for FND might represent a compensatory or stress-buffering upregulation for this subsample specifically. For example, when considering the anxiolytic and stress-buffering effects of the oxytocinergic system, higher peripheral OXT may reflect maladaptive chronic stress-related engagement in some individuals, but compensatory or resilience-related upregulation in others. The present cross-sectional data that were taken from a specific subpopulation of patients with FND and matched HCs cannot distinguish between these possibilities.

### 4.7 Limitations

The main limitation revolves around our limited sample size: The main aim of the study population (and thus sample size) was the investigation of peripheral markers of OXT and its involvement in interoceptive measures. Consequently, our sample size is not powered enough for a genetic assessment. To address this limitation, we conducted a simulation-based post-hoc power analysis for the additive rs53576 model, using the observed genotype distribution and the observed additive effect size. This showed that the observed additive association corresponded to an odds ratio of OR = 1.52. Yet, based on 5000 simulations, the estimated power to detect an effect of this magnitude in our current sample size was approximately 28%. Simulations further indicated that approximately N = 400 participants would be required to achieve 80% power for an additive effect of the observed magnitude, whereas with the current sample size the study would only reach 80% power for substantially larger effects of approximately OR ≥ 2.5. Thus, the genetic findings should be interpreted as exploratory and hypothesis-generating rather than confirmatory. However, due to the knowledge of interaction in the (epi)genetic and peripheral oxytocinergic system, we nonetheless wanted to test the involvement of (epi)genetic markers in the established associations. Further, we wanted to test the potential group differences or odd's ratio of allelic distribution of the *OXTR*, to discuss previously established findings in another similar, but different sample [9,36]. We thus want to highlight that this manuscript should be considered as a relevant and important contribution towards the understanding of the oxytocinergic system in FND, but rather in a descriptive nature, aiming to help build more knowledge and more interest in this approach towards FND. This is further because we did not also globally correct for family-wise analyses for multiple comparisons and considered our analyses rather exploratory. Another relevant limitation concerns the source of biological samples and comparability between samples and their indications.

## 5 Conclusion

In conclusion, we found no evidence for simple group-level differences in *OXTR* rs53576 genotype or *OXTR* methylation in FND, and oxytocinergic genetic and epigenetic markers did not improve the explanation of interoceptive dysfunction. These null findings are important, as they argue against a straightforward oxytocinergic genetic or epigenetic risk profile for FND, while also highlighting the need for larger and adequately powered analyses to confirm these preliminary results. However, the preliminary identified interactions on salivary OXT (i.e. between genotype and intron 1 methylation, as well as between genotype and group) could indicate the relevance of the dynamic oxytocinergic system. While the former has been identified in previous analyses, the latter requires independent replication in larger studies. Hence, we support the addition of genetics, epigenetics, and peripheral markers when attempting to identify the oxytocinergic system as an involved underlying mechanism of a stress-related disorder, such as FND. Similarly, we identified that promoter methylation and salivary OXT contributed to a better model fit for explaining the variance in self-reported childhood trauma. While this represents an interesting and novel finding, given our study design and implemented methods, our interpretation is also constrained to this explorative association that further warrants research to explore a potential interaction-based and stress-related oxytocinergic mechanisms in FND. Together, these preliminary findings also argue against simple (epi)genetic risk effects in FND, but highlighting the importance of considering oxytocinergic interactions in relation to stress and trauma, in future studies.

## Data availability

The R-script markdown used to generate the output for this publication is publicly available on github: https://github.com/FND-ResearchGroup/OXT_FND_NS/tree/main and (genetic) data from participants who signed consent for further use can be requested by the authors.

## Declaration of generative AI and AI-assisted technologies in the manuscript preparation process

During the preparation of this work the authors used ChatGPT and DeepL Write in order to edit the scientific language and aid in statistical analysis. After using this tool/service, the authors reviewed and edited the content as needed and take full responsibility for the content of the published article.

## Funding

This work was supported by the Swiss National Research Foundation Professorship Grant PP00P3_176985.

## CRediT authorship contribution statement

**Natascha Stoffel:** Conceptualization, Data curation, Formal analysis, Investigation, Methodology, Project administration, Visualization, Writing – original draft, Writing – review & editing. **Juan Ansede-Bermejo:** Methodology, Writing – original draft, Writing – review & editing. **Cristina Concetti:** Project administration, Writing – review & editing. **Ángel Carracedo:** Methodology, Supervision, Writing – review & editing. **Selma Aybek:** Funding acquisition, Supervision, Writing – review & editing.

## Declaration of competing interests

The authors declare that they have no known competing financial interests or personal relationships that could have appeared to influence the work reported in this paper.
