# Supplementary material for: The oxytocinergic system in functional neurological disorder: Preliminary testing of associations with interoception and childhood trauma
Source: Compr Psychoneuroendocrinol. 2026 Aug 8;27:100376. doi: 10.1016/j.cpnec.2026.100376 (PMC13487256; doi:10.1016/j.cpnec.2026.100376)
Supplement: Supplementary file 1 [file mmc1.docx]

**The Oxytocinergic System in Functional Neurological Disorder:
Preliminary Testing of Associations with Interoception and Childhood Trauma**

Natascha Stoffel,^a^ Juan Ansede-Bermejo^b,c^, Cristina Concetti,^a^

Ángel Carracedo^b,d^, Selma Aybek^a^

**Author affiliations:**

^a^Faculty of Science and Medicine, University of Fribourg, 1700 Fribourg, Switzerland.

^b^Fundación Pública Galega de Medicina Xenómica, Santiago de Compostela, Spain.

^c^Centro Nacional de Genotipado, Santiago de Compostela, Spain.

^d^Universidade de Santiago de Compostela, Santiago de Compostela, Spain.

**Overview of different study samples**

For better clarity on which publication derived from which study cohort, we list here the publications related to the oxytocinergic system and refer to the study population with their study registration number, while also listing the effective sample size that was available for the specific analysis that has been published, with the main findings or study aims as demonstrated.

| Supplementary Table 1 : Overview of Study samples that discuss relevant aspects | | | |
| --- | --- | --- | --- |
| **Study population** | **Effective sample size** | **Main findings / study aims** | **References** |
| *CER 14-008* | N_FND_ = 15 vs N_HC_ = 16 | Increased *OXTR* intron methylation in FND | (Apazoglou et al., 2018) |
| Swiss cantonal ethical registration SNCTP000002289 | N_FND_ = 85 vs N_HC_ = 76 | Identifying the A allele with higher Odds Ratio for FND in the recessive, genetic model of rs53576 *OXTR* | (Weber et al., 2025a) |
|  | N_FND_ = 59 vs N_HC_ = 65 | Exploring the interaction between genotype, intron methylation, salivary OXT and functional connectivity | (Weber et al., 2025b) |
| Clinicaltrials.gov NCT06084325 | N_FND_ = 43 vs N_HC_ = 48 | Finding decreased respiratory sensitivity in FND, intact metacognition but reduced drift rate for FND | (Stoffel et al., 2025a) |
|  | N_FND_ = 42 vs N_HC_ = 45 | Demonstrating increased salivary OXT in FND, correlation to IAS and group specific interaction with attachment | (Stoffel et al., 2025b) |
|  | N_FND_ = 44 vs N_HC_ = 48 | Discussing altered Heartbeat-evoked potentials in patients with FND |  |
|  | N_FND_ = 37 vs N_HC_ = 42 | Exploring altered predictive coding in FND |  |
|  | N_FND_ = 41 vs N_HC_ = 48 | Testing the three way interaction in oxytocinergic system, and identifying the relevance of dynamic oxytocinergic variables for childhood trauma | PRESENT STUDY |

**Sequence of PCR and SBE primers employed in genotyping assays**

A pool of PCR primers was made at a final concentration of each primer of 100nM. PCR reactions were set up in a final reaction volume of 5 µL and contained 2 µL of template DNA, 1.2 µL of HPLC-grade water, 0.5 µL of PCR buffer (10x), 0.5 µL of MgCl_2_ (25nM), 0.1 µL of dNTPs (25nM), 0.5 µL of pool PCR primers and 0.2 µL of *PCR* enzyme (5U/µL). The thermal cycling conditions for the reaction consisted of an initial denaturation step at 94°C for 2 minutes, followed by 45 cycles of 94°C for 30 seconds, 56°C for 30 seconds and 72°C for 1 minute, followed by a final extension step of 72°C for 1 minute. PCR products were treated with 2µl of SAP cocktail containing 1.53µL of HPLC-grade, 0.17 µL of SAP buffer (10x) and 0.3 µL of shrimp alkaline phosphatase (SAP; 1.7U/µL) by incubation at 37°C for 40 min, followed by enzyme inactivation by heating at 85°C for 5 min to neutralize unincorporated dNTPs.

An extend primer mix was prepared at a final concentration ranged from 0.73 µM to 1.57 µM. An iPLEX extension cocktail was prepared adding 1.2 µL of HPLC-grade water, 0.2 µL of iPLEX buffer (10x), 0.2 µL of iPLEX termination mix, 0.94 µL of extend primer mix and 0.14 µL of iPLEX enzyme (32U/µL). Finally, the iPLEX Gold reactions were set up in a final volume of 9 µL volume adding 2 µL of iPLEX extension cocktail into each well of the reaction plate. The thermal cycling conditions for the iPLEX reaction included an initial denaturation step at 94°C for 30 seconds, followed by 40 cycles of 94°C for 5 seconds, with an internal 5 cycles loop at 52°C for 5 seconds and 80°C for 5 seconds, followed by a final extension step of 72°C for 3 minutes. iPLEX Gold reaction products were desalted employing a clean resin following the manufacturer’s protocol (Agena Bioscience). Desalted products were dispensed onto a 384 SpectroChip II^TM^ using the MassARRAY system with Chip-Prep Module (CPM) and spectra were acquired using the MA4^TM^ mass spectrometer (Agena Bioscience, San Diego, CA), followed by manual inspection of spectra by trained personnel using MassARRAY Typer software v5.0 (Agena Bioscience, San Diego, CA). All assays were conducted in 384-well plates, and as quality control included negative controls and a trio of Coriell samples (Na10861, Na11995 and Na11994) as positive controls.

**Supplementary Table 2: Sequence of PCR and SBE primers employed in genotyping assays**

| **SNP** | **Forward PCR primer** | **Reverse PCR primer** | **SBE primer** |
| --- | --- | --- | --- |
| rs53576 | ACGTTGGATGTGGAAAGGAAAGGTGTACGG | ACGTTGGATGGTAGAATGAGCTTCCCAGCC | TTTCTGTGGGACTGAGGA |
| rs2254298 | ACGTTGGATGATTCAGAGGAAGAAGCCCCG | ACGTTGGATGGCCTGAACAGCTGAACATGG | GAAACCATCCCTGTTTTC |

**Sequence of PCR primers employed in methylation assays**

Reverse primers were tagged with T7 promoter, and 10mer-tag sequence was added to the forward primer for balancing the PCR primers melting temperature.

**Supplementary Table 3. Sequence of PCR primers employed in methylation assays**

| **OXTR Gene Region** | **Coordinates** | **Forward PCR primer** | **Reverse PCR primer** |
| --- | --- | --- | --- |
| Promoter | chr3:8,811,281-8,811,651, | TTTTTTTTAGTATTGTTTAGAATAGAGGTT | TTAATCCCAAAACCTTAACAAAT |
| Intron | chr3:8,810,749-8,810,920 | GTAGGTAGTTGGGTGTTAAGTAGGG | AATCAAAAACTCTAAATCTCAATCCC |

* Reference genome version: GRCh37/hg19. Forward tag sequence: aggaagagag; Reverse tag sequence: cagtaatacgactcactatagggagaaggct.

### **iPLEX Genotyping Assay Overview (Williams et al., 2020)**

Extracted genomic DNA is subjected to multiplexed PCR to amplify regions containing SNPs of interest. After the removal of unincorporated deoxynucleoside triphosphates (dNTPs), a primer of unique known mass anneals immediately adjacent to each SNP and a polymerase catalyzes the addition of the complementary dideoxynucleotide to the SNP as a single-base extension reaction. The addition of this single nucleotide increases the mass of the primer such that the mass spectrometer can resolve the allele-specific extension product. MALDI-TOF MS analysis ionizes and transports the products of the single-base extension reaction to the instrument detector and mass is determined by time of flight. The resultant spectra show ion peak locations for the mass of the unextended primer (UEP) and the allelic extension products with the delta mass from UEP indicating the added nucleotide. A high-quality homozygous call will have a single extended primer peak while a heterozygous call will have 2 peaks of approximately equal height.

### **EpiTYPER Methylation Assay Overview (Freire-Aradas et al., 2016)**

The Agena Bioscience EpiTYPER assay (San Diego, CA, USA) is a bisulfite-treatment-based method for detection and quantitation of DNA methylation (Ehrich *et al.*, 2005). The method starts with bisulfite treatment of genomic DNA, this treatment converted non-methylated Cytosine (C) into Uracil (U). Next by PCR amplification of target regions employing a reverse primer which contains a T7 promoter tag. Next, in vitro RNA transcription is performed, followed by base-specific RNA cleavage. Finally, the cleavage products are analyzed using MALDI-TOF mass spectrometry

Briefly, bisulfite treatment converted non-methylated Cytosine (C) into Uracil (U), including also a PCR amplification using 7T-promoter tag included into the sequence of reverse primer. SAP treatment and followingly in vitro RNA transcription was performed on the reverse strand which contain the T7-promoter sequence, followed by a U-specific cleavage. Using MALDI-TOF, mass spectrometry analysis produced methylation-dependent signal patterns corresponding to the mass difference due to the sequence variation induced by the effect of bisulfite treatment on CpG methylation status.

As result of bisulfite treatment of genomic DNA non-methylated cytosine is converted to uracil and methylated cytosine remains unchanged. Bisulfite treatment produces methylation-dependent sequence variations of C to T. These C/T variations appear as G/A changes on the reverse strand, which result in a mass difference of 16 Da per CpG site in the cleavage products having CpG sites. This mass difference is easily detected by the MassARRAY® system and readily apparent in the signal pattern (Ehrich and von den Boom, 2005).

So, this produces methylation dependent sequence variation of C to T in PCR amplified products. These in turn, appear as G or A variation in the cleavage products generated from the reverse strand by base-specific cleavage, resulting in a mass difference of 16 Daltons (Da) per CpG position, detected by MassARRAY4 (Agena Bioscience, San Diego, CA, USA) mass spectrometry. In the mass spectrum the relative amount of methylation can be calculated by comparing the signal intensity between the mass signals of methylated and non-methylated DNA. As DNA fragments of different mass are required, those of identical size or outside a mass window of 1700–7000 Da cannot be detected and thus fail to give methylation data. In suitably sized DNA fragments MassARRAY4 detects methylation levels as CpG sets, comprising one or multiple CpG positions in the same cleavage fragment.

**Salivary Oxytocin by genotype and group**

| **Supplementary Table 4: Salivary Oxytocin by genotype and group** | | | |
| --- | --- | --- | --- |
| **Group** | **Genotype  rs53576** | **N** | **OXT mean (sd)** |
| HC | AA | 4 | 5.72 (0.85) |
|  | GA | 21 | 7.03 (3.52) |
|  | GG | 22 | 7.23 (3.20) |
| FND | AA | 7 | 10.51 (3.06) |
|  | GA | 17 | 6.48 (2.56) |
|  | GG | 16 | 9.73 (3.74) |

**Recessive model of rs53576 (replication of (Weber et al., 2025a))**

| **Supplementary Table 4. Recessive Model of rs53576** | | | | | | |
| --- | --- | --- | --- | --- | --- | --- |
| **SNP ID** | **Model** | **Genotype** | **Cases** | |  |  |
|  |  |  | **FND,**  **N = 41 (46%)** | **Controls,**  **N = 48 (54%)** | **OR  [95% CI]** | ***P*-value** |
| rs53576 | Recessive | G/G-G/A  A/A | 33 (80.5%)  8 (19.5%) | 44 (91.7%)  4 (8.3%) | 1.00  2.67 [0.74, 9.61] | 0.123 |
| *Abbreviations: OXTR: Oxytocin receptor, OR = Odds Ratio, FND = Functional Neurological Disorder*  *Models were calculated including covariates age, sex, CTQ total and affective sum score. The recessive model assumes dominance of G (either GG or GA) compared to AA phenotype.* | | | | | | |

**Sensitivity Analyses in medication-free sample**

| **Supplementary Table 5. Description of medication-free subsample** | | | | |
| --- | --- | --- | --- | --- |
| **Variable** | **Overall, N=66** | **HC,  N=46** | **FND,  N=20** | **p-value** |
| Sex: count female (%) | 47.0 (71.2) | 33.0 (71.7) | 14.0 (70.0) | >0.9 |
| Age: mean years (SD) | 37.5 (12.6) | 37.5 (13.1) | 37.5 (11.6) | >0.9 |
| Depression: BDI-II median score (IQR) | 6.0 (8.8) | 4.0 (7.0) | 11.0 (11.3) | <0.001 |
| Trait Anxiety: STAI-T mean score (SD) | 38.7 (10.4) | 37.2 (10.5) | 42.1 (9.4) | 0.068 |
| Childhood Trauma:  CTQ median score (IQR) | 34.5 (19.0) | 33.5 (10.5) | 43.5 (20.0) | 0.024 |

Testing the group difference in methylation and the three-way interaction in the non-medicated subpopulation as a sensitivity analysis yielded no significant results. For the average intron region W = 349, p = 0.121 and for the promoter region W = 492, p = 0.665.

Concerning the three-way interaction models including salivary OXT, rs53576 genotype, and *OXTR* methylation did not retain the significant main effects or two-way interactions observed in the full sample. However, in the model with the intron region, the two-way interaction between group and rs53576 genotype remained at trend level, F(2,52) = 3.16, p = 0.051. This effect was attenuated after adjusting for age and affective symptoms, F(2,50) = 2.91, p = 0.064. For the model with the promoter región, the group × rs53576 genotype interaction remained at trend level in the unadjusted model, F(2,52) = 2.94, p = 0.062, and was again attenuated after adjustment for age and anxiety/depression symptom burden, F(2,50) = 2.82, p = 0.069. Overall, these sensitivity analyses suggest that the identified genotype-dependent group differences in salivary OXT were directionally consistent but weaker in the medication-free subgroup, raising the question about the involvement of medication in these results, but also noting that this sensitivity analysis has even a lower sample size that is also unbalanced between groups.

**Sex-Stratified Analyses for *OXTR***

As an additional sensitivity analysis for the *OXTR* promoter region with known sex-stratified effects (Gouin et al., 2017), we tested the promoter region in the female population only. There was no difference between the groups W = 515, p = 0.767 and we did not identify any significant main or interaction effects in the three-way interaction model. Specifically, there was no significant main effect of group, F(1,51) = 1.07, p = 0.306, rs53576 genotype, F(2,51) = 2.01, p = 0.144, or *OXTR* promoter methylation, F(1,51) = 1.73, p = 0.195. The group × rs53576 genotype interaction was also not significant, F(2,51) = 1.53, p = 0.226, nor was the three-way interaction between group, rs53576 genotype, and *OXTR* promoter methylation, F(2,51) = 0.34, p = 0.713. Thus, also in the female-only subgroup, *OXTR* promoter methylation did not seem to moderate the association between group, rs53576 genotype, and salivary OXT.

**Visualization of Interoceptive with oxytocinergic variables**

For the two interoceptive variables further assessed in this study (behavioural outcome variable from respiratory resistance sensitivity task; RRST and self-report on interoceptive accuracy scale; IAS), as well as for the self-report on childhood trauma; CTQ, Spearman rank correlation analyses were conducted separately by group to visualize the associations with oxytocin-related measures. Specifically, each interoceptive variable was correlated with average *OXTR* intron and promoter methylation levels, and salivary oxytocin concentrations. Scatterplots were generated with group-specific regression lines and confidence intervals to illustrate the direction of these associations. Correlation coefficients and corresponding p-values were displayed separately for each group.

**SUPPLEMENTARY REFERENCES**

Apazoglou, K., Adouan, W., Aubry, J.-M., Dayer, A., Aybek, S., 2018. Increased methylation of the oxytocin receptor gene in motor functional neurological disorder: a preliminary study. J. Neurol. Neurosurg. Psychiatry 89, 552–554. https://doi.org/10.1136/jnnp-2017-316469

Ehrich, M., von den Boom, D., 2005. Introduction to EpiTYPER for Quantitative DNA Methylation Analysis Using the MassARRAY System.

Freire-Aradas, A., Phillips, C., Mosquera-Miguel, A., Girón-Santamaría, L., Gómez-Tato, A., Casares de Cal, M., Álvarez-Dios, J., Ansede-Bermejo, J., Torres-Español, M., Schneider, P.M., Pośpiech, E., Branicki, W., Carracedo, Á., Lareu, M.V., 2016. Development of a methylation marker set for forensic age estimation using analysis of public methylation data and the Agena Bioscience EpiTYPER system. Forensic Sci. Int. Genet. 24, 65–74. https://doi.org/10.1016/j.fsigen.2016.06.005

Gouin, J.P., Zhou, Q.Q., Booij, L., Boivin, M., Côté, S.M., Hébert, M., Ouellet-Morin, I., Szyf, M., Tremblay, R.E., Turecki, G., Vitaro, F., 2017. Associations among oxytocin receptor gene (OXTR) DNA methylation in adulthood, exposure to early life adversity, and childhood trajectories of anxiousness. Sci. Rep. 7, 7446. https://doi.org/10.1038/s41598-017-07950-x

Stoffel, N., Sojka, P., Gninenko, N., Mouthon, M., von der Weid, L., Serranová, T., Aybek, S., 2025a. Respiratory sensitivity is reduced in functional neurological disorder and associated with higher somatoform dissociation. Brain Commun. 7, fcaf283. https://doi.org/10.1093/braincomms/fcaf283

Stoffel, N., von der Weid, L., Gross, J., Concetti, C., Bruckmaier, R., Aybek, S., 2025b. Increased salivary Oxytocin correlates with lower self-reported Interoceptive Accuracy in Functional Neurological Disorders. Neurobiol. Stress 100765. https://doi.org/10.1016/j.ynstr.2025.100765

Weber, S., Álvarez, L.T.R., Stoffel, N., Ansede-Bermejo, J., Cruz, R., Del Real Bolt, Á., Bühler, J., Carracedo, Á., Aybek, S., 2025a. Increased Prevalence of an Oxytocin Receptor Gene Variant in Functional Neurological Disorder: A Case-Control Study. J. Neuropsychiatry Clin. Neurosci. appi.neuropsych.20250031. https://doi.org/10.1176/appi.neuropsych.20250031

Weber, S., Stoffel, N., Ansede-Bermejo, J., Cruz, R., Del Real Bolt, Á., Bruckmaier, R., Carracedo, Á., Aybek, S., 2025b. Salivary oxytocin and amygdalar alterations in functional neurological disorders. Brain Commun. 7, fcae455. https://doi.org/10.1093/braincomms/fcae455

Williams, G.R., Cook, L., Lewis, L.D., Tsongalis, G.J., Nerenz, R.D., 2020. Clinical Validation of a 106-SNV MALDI-ToF MS Pharmacogenomic Panel. J. Appl. Lab. Med. 5, 454–466. https://doi.org/10.1093/jalm/jfaa018
